# Genomic islands and molecular mechanisms relating to drug-resistance in *Clostridioides* (*Clostridium*) *difficile* PCR ribotype 176

**DOI:** 10.1080/22221751.2025.2482698

**Published:** 2025-03-25

**Authors:** Krutova Marcela, Kovarovic Vojtech, Brajerova Marie, Demay Fanny, Zikova Jaroslava, Prasad Suhanya, Soltesova Anna, Novakova Elena, Pituch Hanna, Kuijper Ed, Smits Wiep Klaas, Balikova Novotna Gabriela

**Affiliations:** a Department of Medical Microbiology, Charles University Second Faculty of Medicine and Motol University Hospital, Prague, Czech Republic; b European Society of Clinical Microbiology and Infectious Diseases (ESCMID) study group for *Clostridioides difficile* (ESGCD), Basel, Switzerland; c Institute of Microbiology, The Czech Academy of Sciences, BIOCEV, Vestec, Czech Republic; d Department of Clinical Microbiology, Unilabs Slovakia Inc., Roznava, Slovakia; e Department of Microbiology and Immunology, Comenius University Jessenius Faculty of Medicine in Martin, Martin, Slovakia; f Department of Medical Microbiology, Medical University of Warsaw, Warsaw, Poland; g Dutch National Expertise Centre for *Clostridioides difficile* infections, Leiden University Center for Infectious Diseases, Leiden and Centre for Infectious Disease Control (CIb), National Institute for Public Health and the Environment (RIVM), Bilthoven, Netherlands; h Experimental Bacteriology, Leiden University Center for Infectious Diseases, Leiden Medical Center, Leiden, Netherlands

**Keywords:** *Clostridioides difficile* infection, epidemiology, macrolide resistance methyltransferase, whole genome sequencing

## Abstract

**Objectives:**

To analyse characteristics of *Clostridioides difficile* PCR ribotype 176 clinical isolates from Poland, the Czech Republic and Slovakia with regard to the differences in its epidemiology.

**Methods:**

Antimicrobial susceptibility testing and whole genome sequencing were performed on a selected group of 22 clonally related isolates as determined by multilocus variable-number tandem repeat analysis (n = 509). Heterologous expression and functional analysis of the newly identified methyltransferase were performed.

**Results:**

Core genome multilocus sequence typing found 10–37 allele differences. All isolates were resistant to fluoroquinolones (*gyr*A_p. T82I), aminoglycosides with *aac*(6’)-*Ie-aph*(2'’)-*Ia* in six isolates. Erythromycin resistance was detected in 21/22 isolates and 15 were also resistant to clindamycin with *erm*B gene. Fourteen isolates were resistant to rifampicin with *rpo*B_p. R505K or p. R505K/H502N, and five to imipenem with *pbp*1_p. P491L and *pbp*3_p. N537K. P*nim*B^G^ together with *nim*B_p. L155I were detected in all isolates but only five were resistant to metronidazole on chocolate agar. The *cfr*E, *van*Z1 and *cat*-like genes were not associated with linezolid, teicoplanin and chloramphenicol resistance, respectively. The genome comparison identified six transposons carrying antimicrobial resistance genes. The *erm*B gene was carried by new Tn*7808*, Tn*6189* and Tn*6218*-like. The *aac*(6’)-*Ie-aph*(2'’)-*Ia* were carried by Tn*6218*-like and new Tn*7806* together with *cfr*E gene. New Tn*7807* carried a *cat*-like gene. Tn*6110* and new Tn*7806* contained an RlmN-type 23S rRNA methyltransferase, designated MrmA, associated with high-level macrolide resistance in isolates without *erm*B gene.

**Conclusions:**

Multidrug-resistant *C. difficile* PCR ribotype 176 isolates carry already described and unique transposons. A novel mechanism for erythromycin resistance in *C. difficile* was identified.

## Background

*Clostridioides difficile* is the most common infectious cause of healthcare-associated diarrhoea. An increase in *C. difficile* infections (CDI) was reported in 2003, after the emergence and worldwide spread of *C. difficile* PCR ribotype (RT) 027 [1]. In the latest European study, the five most common RTs from hospitals were 027 (11%; n  =  21), 181 (12%; n  =  24), 014 (8%; n  =  15), 010 (5%; n  =  10), and 002 (5%; n  =  9) but RTs 027, 181 and 176 dominate in the Eastern Europe [2]. These three ribotypes are genetically related, belong to clade 2 and sequence type 1, carry genes for binary toxin, and have an 18 bp deletion at position 311 and a single nucleotide deletion at position 117 in the *tcdC* gene but can be distinguished by capillary electrophoresis ribotyping [3, 4]. In addition, the ECDC surveillance data indicated that RT176 was associated with poor infection outcomes [5].

Interestingly, the differences in the epidemiology of infections caused by *C. difficile* RT176 have been noted between Poland, the Czech Republic and Slovakia, and over time per country. In Poland, the prevalence of RT176 is low, compared to the dominant RT027 (99/166, 62.3% vs 22/166, 13.8% in 2012-2013, 13 hospitals) [6]. In the Czech Republic, the incidence of *C. difficile* RT176 was high (251/624, 40%, 2013, 11 hospitals), but this RT has since been replaced by the currently dominant RT001 (127/379, 33.5% vs 44/379 11.6% in 2017, 16 hospitals) [7,8]. In contrast, RT176 has emerged recently in Slovakia, when RT176 was absent in 10 hospitals in Slovakia in 2012 but has become the dominant PCR ribotype in Slovak acute healthcare in 2018–2019 (185/370, 50%, 14 hospitals) [9,10].

We hypothesized that differences in RT176 epidemiology in individual countries could be related to genomic differences that can provide a selective advantage. Therefore, we investigated the genomic properties of the isolates of this ribotype in detail.

## Material and methods

### MLVA retrospective data analysis for selection of isolates to the study

The isolates for further characterization were selected by intercountry clonal relatedness determined by MLVA using data from previously published studies [10–14]. We included 509 *C. difficile* isolates in total; 267 (52%) isolates from Slovakia (2016-2019; 16 hospitals), 225 (44%) from the Czech Republic (2014; 14 hospitals), and 17 (3%) from Poland (2006 and 2012; 5 hospitals). All isolates were identified as *C. difficile* RT176 by capillary PCR ribotyping and carried *tcd*A-toxin A, *tcd*B-toxin B, *ctd*A and *cdt*B-binary toxin genes, respectively [15, 16]. A minimum spanning tree (MST) using MLVA results of seven previously published regions was generated using Bionumerics v5.0 (Applied Maths). Clonal relatedness was defined as the sum of tandem repeat differences (STRD) ≤ 2 [17].

### Whole genome sequencing

Short-read sequencing was performed for 22 *C. difficile* RT176 isolates selected based on intercountry clonal relatedness from MLVA as described above. Bacterial DNA was extracted using a Qiagen Blood Mini kit (Qiagen, Hilden, Germany), DNA library was prepared by Nextera XT library preparation kit (Illumina, San Diego, California, USA) and sent for whole genome sequencing (WGS) using NovaSeq6000 Illumina (San Diego, California, USA), at Macrogen (Seoul, South Korea). Sequenced genomes were assembled using SPAdes v3.15.5 [18] and annotated in RAST (https://rast.nmpdr.org/).

Subsequently, long-read sequencing was performed for five *C. difficile* isolates selected based on different antimicrobial resistance patterns to determine the precise genomic context of antimicrobial resistance determinants. The bacterial DNA was extracted using MasterPure™ Gram Positive DNA Purification Kit (Biosearch Technologies, Hoddesdon, UK). The DNA library was prepared using a Ligation Sequencing Kit #SQK-LSK109 and sequenced in a MinION flow cell #FLO-MIN106 (Oxford Nanopore Technologies, Oxford, UK). Fast5 read files were base-called and converted to fastQ using Guppy v3.0.3 + 7e7b7d0 (Oxford Nanopore Technologies, Oxford, UK). Hybrid assembly from short and long reads was performed using Flye v2.9.1 (long reads assembly) [19], Medaka v1.7.2 (polishing by long reads) (Oxford Nanopore Technologies, Oxford, UK) and Polypolish v0.5.0 (polishing by short reads) [20]. Data on genome length and coverage are provided in Supplementary material, Table S1. Genomes were annotated in RAST with default settings (https://rast.nmpdr.org/).

### Bioinformatic analysis

A multilocus sequence type (ST) and a core genome multilocus sequence type (cgMLST) were determined from FASTQ data using MLSTFinder v2.0 and cgMLSTFinder v1.1 (https://www.genomicepidemiology.org/). The core genome multilocus sequence typing (cgMLST) was performed and MST was constructed using BioNumerics (v8.1, 1975 loci, bioMérieux, France). The phylogenetic trees were constructed as follows: first, the single nucleotide polymorphisms (SNPs) were called from sequencing reads aligned to reference genome C1174 using CSI phylogeny v1.6.1 [21]. The RAxML-NG v1.2.2 was then used to infer the Maximum-Likelihood tree with the GTR + G model and 500 bootstrap replicates. Finally, iTOL v6.9.1 was used to visualize this phylogenetic tree [22, 23].

Antimicrobial resistance genes were predicted with Abricate software v1.0.1 [24] using the CARD database (https://card.mcmaster.ca/analyze/rgi) and acquired antimicrobial resistance genes were detected also using ResFinder v4.1 after the upload of FASTQ data, default setting and “selected species other” (https://www.genomicepidemiology.org/) and then identified in annotated assemblies. Mutations in the *gyr*A gene resulting in amino acid substitution T82I associated with fluoroquinolone resistance and in the *rpo*B gene resulting in amino acid substitutions associated with rifampicin resistance, mutations in gene encoding PBPs resulting in amino acid substitutions associated with carbapenem resistance [1,25] and mutations in the *hsm*A gene and the *nim*B gene and its promotor associated with metronidazole resistance [26,27] were searched by alignment (Geneious software v2021.0.3) to *Clostridioides difficile* 630 genome (GCF_000009205.2). The presence of pCD-METRO [28] was searched by mapping short reads to the metronidazole-resistant *C. difficile* RT020, IB136; GCF_900696735.1. The whole genome and the linear comparisons were visualized using EasyFig v2.2.5 [29] to identify the genomic context of antimicrobial resistance determinants. Sequence similarity between genomic regions of interest was determined using Blastn with a maximum e-value of 0.001 and no minimum identity values. The amino acid sequences that are part of the identified inserts were compared to the UniProt database. The presence of identified inserts was searched in the remaining sequenced *C. difficile* isolates by mapping short reads to complete genomes from this study using Geneious v2021.0.3, verified by blastn searches against sequenced genomes and visualized both using CLC Main Workbench version v24.0 (QIAGEN, Hilden, Germany, Aarhus A/S) with modifications using Inkscape v1.1. Other mobile genetic elements were predicted by IslandViewer 4 [30] MobileElementFinder v1.0.3 [31] and the ICEfinder tool from the ICEBerg v3.0 database [32]. The predictions were manually curated, annotated and visualized in the genome alignment with EasyFig v2.2.5 [29]. Novel transposon numbers were obtained from the Transposon Registry [33].

The integrative and conjugative transposons were also searched in the available 54 sequences of RT176 from previously published studies [10, 34] and the unrooted Maximum-Likelihood phylogenetic tree was constructed as described above.

### Antimicrobial susceptibility testing

Minimum inhibitory concentration (MIC) to 19 antimicrobials (see below) was tested by an antimicrobial concentration gradient testing strip (E-test, Liofilchem®, Roseto degli Abruzzi, Italy, individual range is provided in Supplementary material, Table S2) on Schaedler agar with sheep blood, haemin and vitamin K1 (Oxoid, Basingstoke, UK). In addition, MIC to fidaxomicin was evaluated by agar dilution method on Wilkins Chalgren agar (Oxoid, Basingstoke, UK) in duplicates and susceptibility to metronidazole was tested using E-test on Fastidious Anaerobe agar supplemented with 5% horse blood (Oxoid, Basingstoke, UK) and chocolate agar with Vitox supplemented with a defined growth supplement and haemoglobin (Oxoid, Basingstoke, UK). Plates were inoculated by bacterial suspension of 1 McFarland and cultured for 48 hours in an anaerobic atmosphere at 36.6°C (Anaerobic Workstations, Don Whitley Scientific, UK).

The European Committee on Antimicrobial Susceptibility Testing (EUCAST) epidemiological cut-off values (ECOFFs) were applied for metronidazole (>2 mg/L), vancomycin (>2 mg/L) and fidaxomicin (>0.5 mg/L), (v13.1). The Clinical and Laboratory Standards Institute (CLSI) breakpoints were used for tetracycline (≥16 mg/L), ciprofloxacin and moxifloxacin (≥8 mg/L), clindamycin (≥8 mg/L), erythromycin (≥8 mg/L), linezolid (≥4 mg/L), amoxicillin (≥16 mg/L), carbapenems (meropenem, imipenem and ertapenem, ≥ 16 mg/L) (30th edition). Due to the absence of breakpoints or ECOFFs for following antimicrobials in *C. difficile*, the EUCAST breakpoints for *Staphylococcus aureus* were used for tigecycline (>0.5 mg/L), rifampicin (>0.06 mg/L), amikacin (>16 mg/L) and gentamicin (>2 mg/L). *Bacteroides fragilis* ATCC 25285 was used as a metronidazole-susceptible control and a previously described *C. difficile* RT010 (metronidazole MIC 12 mg/L) [35] as a metronidazole-reduced susceptibility control.

### Construction of plasmid for inducible macrolide resistance methyltransferase A (mrmA) expression in Escherichia coli

To facilitate inducible expression of the *C. difficile mrm*A gene in *E. coli*, a recombinant plasmid was constructed. The *mrm*A gene was PCR-amplified from genomic DNA (*C. difficile* RT176 isolate 562) using primers: XbaI-6his-Mrm(A) Forward (5’-ACGTTCTAGAAAGGAGATATACCATGCATCATCATCATCATCATAAACGTTTACCTAAATATAC-3’) and Mrm(A)-HindIII Reverse (5’-TGCAAAGCTTT CATTTTTTTTTGAATTTATTAT-3’). This strategy incorporated a six-histidine tag at the N-terminus of the protein, enabling subsequent metal affinity chromatography purification and confirmation of MrmA by mass spectrometry. Importantly, previous studies have demonstrated that this modification does not compromise the activity of homologous proteins [36–38]. The amplified fragment was then directionally cloned into the pBAD30 vector using XbaI and HindIII restriction sites. The resulting recombinant plasmid p6hisMrmA, was verified by Sanger sequencing for accuracy.

### Induction of the mrmA gene expression and susceptibility testing

Efflux deficient BW25113 Δ*tolC* reference JW5503-1 from Keio collection; [39] was used because it is sensitive to macrolides. The BW25113 Δ*tolC* transformed with either p6hisMrmA or the empty pBAD30 vector were grown overnight in LB agar supplemented with 0.2% arabinose to induce the *mrm*A gene expression. Then, inocula were prepared by diluting overnight cultures with sterile saline (0.9% NaCl) to a 1 McFarland turbidity standard. Then, five microliters of each inoculum were mixed with 100 µl of LB broth containing 0.2% arabinose and a two-fold serial dilution of antimicrobials. The plates were incubated at 37°C for 24 hours, and bacterial growth was assessed by measuring the optical density at 600 nm (BIOTEK, Synergy HT, Agilent, Santa Clara, California, USA). MIC to erythromycin was defined as the lowest drug concentration that resulted in at least 80% growth inhibition compared to the no antimicrobial control. All experiments were carried out independently in duplicate, each with three technical replicates. The susceptibility to linezolid and quinupristin/dalfopristin was determined using the E-test method (LIN 0.016-256 µg/mL, REF 412396, bioMérieux, Marcy-l'Étoile, France). Agar dilution assays were performed to assess susceptibility to erythromycin (REF E122.0025, Duchefa Biochemie, Haarlem, The Netherlands), tylosin (REF PHR2652-500 mg, Sigma-Aldrich, Burlington, Massachusetts, USA), chloramphenicol (REF C0123.0025, Duchefa Biochemie, Haarlem, The Netherlands), clindamycin hydrochloride (REF PHR1159-1 g, Sigma-Aldrich, Burlington, Massachusetts, USA), pristinamycin IA and pristinamycin IIA (kindly provided by Aventis Pharma, Vitry-sur-Seine, France). Carbenicillin disodium salt (REF 195092, MP Biomedicals, Navi Mumbai, India) was used for plasmid selection on LB agar.

In addition, to confirm the production of MrmA in *E. coli* BW25113 Δ*tolC*, we purified MrmA fused to an N-terminal His-tag on a small scale as follows. The cell pellet of 0.2% arabinose-induced or non-induced cultures was washed with 1 mL PBS and resuspended in ice-cold Soluble Lysis Buffer (50 mM Tris-HCl pH 8.0, 300 mM NaCl, 10 mM imidazole, 10% glycerol). The bacterial suspensions were lysed with beads (Fast-Prep-24, MPbiomedicals) and centrifuged at 12 000 g for 15 min at 4 °C. The clarified supernatant was collected as a supernatant. Then the remaining pellet was resuspended in insoluble lysis buffer (8M urea, 50 mM Tris-HCl pH 8.0, 300 mM NaCl, 10 mM imidazole). This insoluble fraction was incubated for 30 minutes at room temperature with slow agitation and then centrifuged for 15 minutes at 12 000 g at 4 °C. The soluble and insoluble fractions from the induced and non-induced cultures were incubated for 90 min with Ni-INDIGO beads (PureCube 100 Ni-INDIGO Agarose, Cube BioTech) pre-equilibrated with the lysis buffer. The beads were washed twice with the wash buffer (20 mM imidazole) and finally eluted with (500 mM imidazole, 50 mM Tris-HCl pH 8.0, 300 mM NaCl, 500 mM imidazole) with an additional 8M urea to elute the protein from the insoluble fraction. The input and elution of each sample were analyzed on a 12.5% SDS side gel to detect the band corresponding to MrmA (40 kDa), which was then analyzed by LC-MS for protein identification*.*

## Results

*C. difficile* isolates of RT176 were selected by intercountry clonal relatedness determined by MLVA from retrospective data. Using a cut-off of 2 STRDs, MLVA identified six intercountry clonal complexes (CC 1-6). CC1 and CC2 contained isolates from all three countries, CC3 and CC6 included isolates from the Czech Republic and Slovakia, CC4 had isolates from Poland and Slovakia and CC5 was formed by isolates from the Czech Republic and Poland (Figure S1). A total of 22 *C. difficile* isolates were selected for antimicrobial susceptibility testing and WGS (Figure S1, black arrows; Slovak n = 10, Czech n = 6; Polish n = 6).

All sequenced isolates (n = 22) belonged to sequence type (ST) 1 in Clade 2. According to the cgMLSTFinder, 18 isolates were designated as type 301, two isolates as type 6593 and two isolates as types 4842 and 6493, respectively (Supplementary data Table S3). Using the Bionumerics scheme, cgMLST found a 10–37 allele difference between isolates (Figure S2) which is greater than the proposed adjusted threshold of zero to three allelic differences for cluster relatedness [4].

All tested *C. difficile* isolates were susceptible to vancomycin (geometric mean MIC 0.3 mg/L, range 0.19-0.5 mg/L), fidaxomicin (geometric mean MIC 0.01 mg/L, range 0.0039-0.03 mg/L), linezolid (geometric mean MIC 1.0 mg/L, range 0.38-2 mg/L, with the *cfr*E gene in five isolates without increased MICs), amoxicillin (geometric mean MIC 0.74 mg/L, range 0.25-1.5 mg/L), meropenem (geometric mean MIC 1.7 mg/L, range 0.75-4 mg/L), ertapenem (geometric mean MIC 4.5 mg/L, range 2.0-12 mg/L) and tetracycline (geometric mean MIC 0.04 mg/L, range 0.03-0.094 mg/L). All tested isolates were also susceptible to metronidazole (geometric mean MIC 0.32 mg/L, range 0.19-2.0 mg/L) on Fastidious anaerobe agar supplemented with 5% horse blood. Analysing genetic metronidazole resistance determinants, no mutations in the *hsm*A gene and no presence of pCD-METRO were found, but all isolates had a T-to-G mutation in the promoter of the *nim*B gene (PnimB^G^), associated with heme-dependent metronidazole resistance [27], together with a mutation resulting in a *nim*B_p.L155I substitution. Re-testing of susceptibility to metronidazole on chocolate agar with Vitox (a hemin-enriched medium) increased the geometric mean MIC to 1.8 mg/L (range 1–4 mg/L), and 5/22 isolates qualified as resistant (MIC >2 mg/L) under these conditions and 17 isolates remained susceptible.

All tested *C. difficile* were resistant to ciprofloxacin and moxifloxacin (geometric mean MIC ≥32 mg/L) with *gyr*A_p.T82I. Furthermore, all isolates were resistant to gentamicin and amikacin (geometric mean MICs of 84.1 and 145.2 mg/L, respectively). Interestingly, isolates carrying *aac*(6’)-*Ie-aph*(2'’)-*Ia* (6/22) showed a higher geometric mean MIC of ≥256 mg/L for both antimicrobials compared to a geometric mean of 55.4 and 117.4 mg/L to gentamicin and amikacin, respectively, in isolates lacking this resistance determinants. Five isolates were resistant to imipenem (≥32 mg/L) with *pbp*1_p.P491L and *pbp*3_p.N537K. The isolates with no amino acid substitutions in PBPs had a geometric mean MIC of 7.8 mg/L (range 6–12 mg/L). 21/22 isolates were resistant to erythromycin (geometric man MIC of 190.4 mg/L, range 0.38-256 mg/L) and 15/22 were also resistant to clindamycin (geometric mean MIC of 52.3 mg/L, range 0.75-256 mg/L) and carried the *erm*B gene. Thus, in six erythromycin-resistant but clindamycin-susceptible isolates, the resistance mechanism was not identified. For that reason, these isolates were further investigated below.

Although no official breakpoints are currently available for tigecycline and rifampicin in *C. difficile*, for tigecycline the geometric mean MIC was 0.05 mg/L (range 0.016-0.12 mg/L), and all isolates can therefore be considered susceptible (in accordance with EUCAST tigecycline breakpoint for *S. aureus* 0.5 mg/L). For rifampicin, there were two groups of isolates. The isolates with a wild-type *rpo*B gene (8/22) displayed a MIC of <0.016 mg/L and the 14 isolates with *rpo*B_p.R505 K or p.R505 K/D492E or p.R505 K/H502N had a geometric mean MIC of 168.8 mg/L (range 48–256 mg/L). We consider the latter isolates to be resistant to rifampicin (in agreement with EUCAST rifampicin breakpoint of >0.06 mg/L for *S. aureus*).

Although the presence of the *van*Z1 gene was detected in all isolates, the geometric mean MIC to teicoplanin was 0.1 mg/L (range 0.064-0.19 mg/L) and to vancomycin was 0.30 mg/L, range 0.19-0.5 mg/L, thus isolates can be considered as susceptible to glycopeptides. On the other hand, the *cat*-like gene was detected in 17/22 isolates but the geometric mean MIC to chloramphenicol was higher in isolates without the *cat*-like gene (7.7 mg/L vs 4.6 mg/L).

A summary of antimicrobial susceptibility testing and the presence of molecular mechanisms of antimicrobial resistance is provided in Supplementary data, Table S2.

A hybrid assembly of long and short reads was constructed for five selected isolates (C562, C1174, S3981, C1478, S4723) each representing a different acquired resistance gene pattern to determine the genomic context of the antimicrobial resistance determinants. Data on genome length and coverage are provided in Supplementary material Table S1. *C. difficile* Czech isolate C1174 was used as the reference genome as it was susceptible to erythromycin.

**Figure 1.**
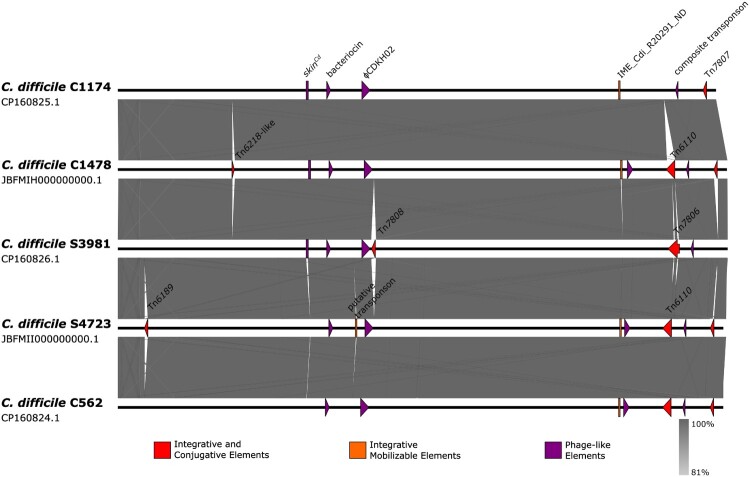
**Whole genome alignment of complete genomes of RT176 *C. difficile***. Nucleotide blast hits shorter than 3000 bp were omitted. Mobile genetic elements are shown and colour-coded as shown in legend.

**Figure 2.**
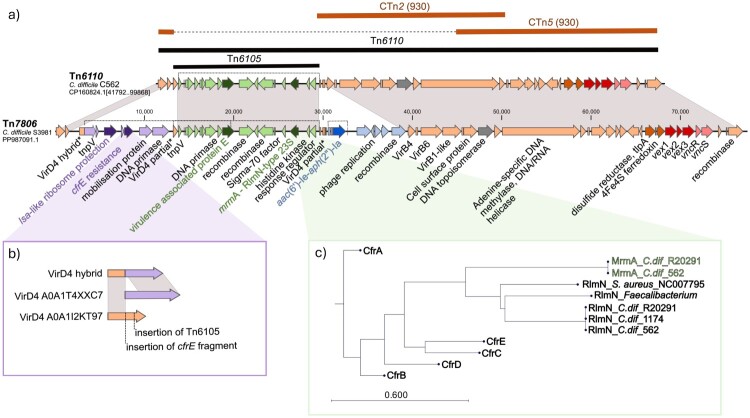
**Schematic representation of the identified Tn*7806* in *C. difficile* isolates and comparison with previously identified conjugative *C. difficile* transposons.** a) Tn*6110*, which belongs to the CTn5-like elements, but also shows similarities with CTn2, both of which have been described in *C. difficile* strain 630. Tn*7806* is identical to Tn*6110* but contains two additional insertions carrying *lsa*-like, *cfr*E and *aac*(6´)-Ib resistance genes, respectively. The dashed line connects different parts of the same gene. b) Detail showing that the gene encoding the T4SS-ATPase VirD4 is disrupted by the insertion of Tn*6105* (in Tn*6110*) and by the additional insertion of the *cfr*E-carrying insert. The insertion of the *cfr*E fragment leads to an in-frame fusion of *virD4* with another *virD4* gene (for details see Figure S4). c) Phylogenetic tree showing the relatedness of the macrolide resistance methyltransferase A (MrmA) identified in Tn*6110* and Tn*7806* to genomic RlmN and antimicrobial resistance Cfr 23S rRNA SAM radical methyltransferases.

The identified acquired resistance genes were localized on integrative and conjugative elements (Figure 1). A 58.1 kb insert corresponding to Tn*6110* (GeneBank BK008009) is present in isolates C562, C1478, and S4723. Tn*6110* is composed of transposon Tn*6105* and a backbone with homology to CTn*2* and CTn*5* from *C. difficile* 630 [40,41], Figure 2a, Supplementary data Table S4.

A highly similar element designated Tn*7806* (77 kb)*,* was found in strain S3981 at the same locus (Supplementary data Table S4). It differs from Tn*6110* by two additional insertions which flank Tn*6105*. The first insertion carries the *lsa*A*-*like gene encoding putative antimicrobial resistance ABCF protein that protects the ribosome [42] and a *cfr*E gene encoding a 23S rRNA methyltransferase. The second insertion carries the *aac*(6’)-*Ie-aph*(2'’)-*Ia* gene for aminoglycoside resistance.

**Figure 3.**
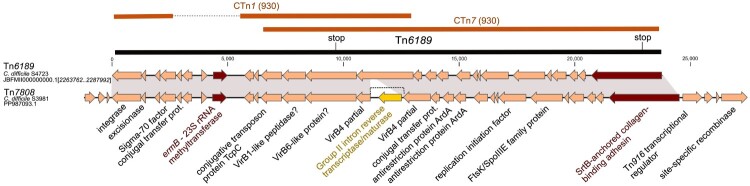
**Schematic representation of the *ermB*-carrying Tn*6189*-derived elements**. Tn*6189*-like and Tn*7808* kb identified in this study are compared with previously identified conjugative *C. difficile* transposons. Tn*6189* shows similarities to CTn*7* and CTn*1* from *C. difficile* strain 630. Nucleotide changes leading to premature stop codons in genes of Tn*6189* are labelled.

We found the erythromycin/clindamycin resistance determinant *erm*B to be carried by three different transposons in this study. A 24.3 kb insert similar to Tn*6189* was found in isolate S4723 (MK895712, Supplementary data Table S4). Isolates S4352 and S4563 have a distinct variant of Tn*6189*, which differs in the chromosomal integration site and a hypothetical gene with a DUF3796 domain and an adjacent gene with a helix-turn-helix domain. *C. difficile* isolate S3981 harbours another 34.2 kb insert named Tn*7808*, which is also similar to the previous Tn*6189* but with an additional 2.7 kb putative intron encoding a retron-type RNA-directed DNA polymerase and extensions at both ends (Figure 3, Supplementary data, Table S4). A BLAST search showed similarity to transposon Tn*5386* (DQ321786.1) from *Enterococcus faecium,* which has an identical putative intron [43]. In the studied set only *C. difficile* isolate C1478 contained a Tn*6218*-like element with *erm*B and *aac*(6’)*-Ie-aph*(2'’)*-Ia* genes encoding ribosomal methylase and bifunctional aminoglycoside modifying enzyme, respectively. This 16.1 kb insert is almost identical to the Tn*6218*-like transposon from the large plasmids pQHY-2 (CP118266) and p21-D-5b (CP119189.1) from *Clostridium perfringens;* it differs only in putative integrase and transposase genes (Supplementary data Table S4).

Further analysis of predicted mobile genetic elements has revealed the presence of one more, Tn*7807* (23.2 kb) similar to CTn*1* [40] designated as Tn*7807*, which harbours the *cat*-like gene and ABC transporter gene cluster. However, based on our susceptibility testing data, no association was found with chloramphenicol resistance and its presence.

**Figure 4.**
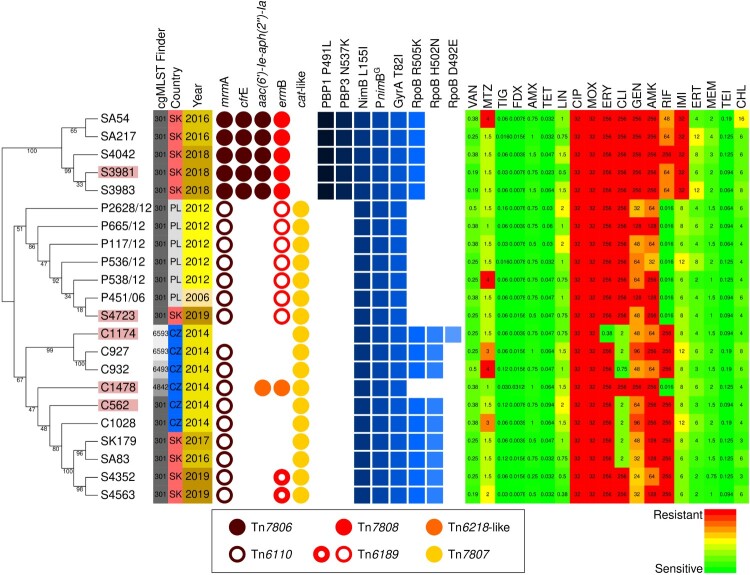
**Unrooted Approximate-Maximum-Likelihood phylogenetic tree inferred with GTR model from core genome alignment (3149673 sites)**. *C. difficile* isolates of PCR ribotype 176 for which complete genomes were generated based on hybrid assembly of Illumina and Nanopore data are highlighted with red background. Country, year of isolation, and cgMLST Finder group are shown for each strain. Circles denote acquired resistance determinants encoded by insert. Related inserts share a colour. Note that Tn*6189* exists in two variants as shown in the legend. Squares show the presence of point mutations conferring resistance. A heatmap of MIC values is shown, and the colour scale is normalized for each respective breakpoint.

**Figure 5.**
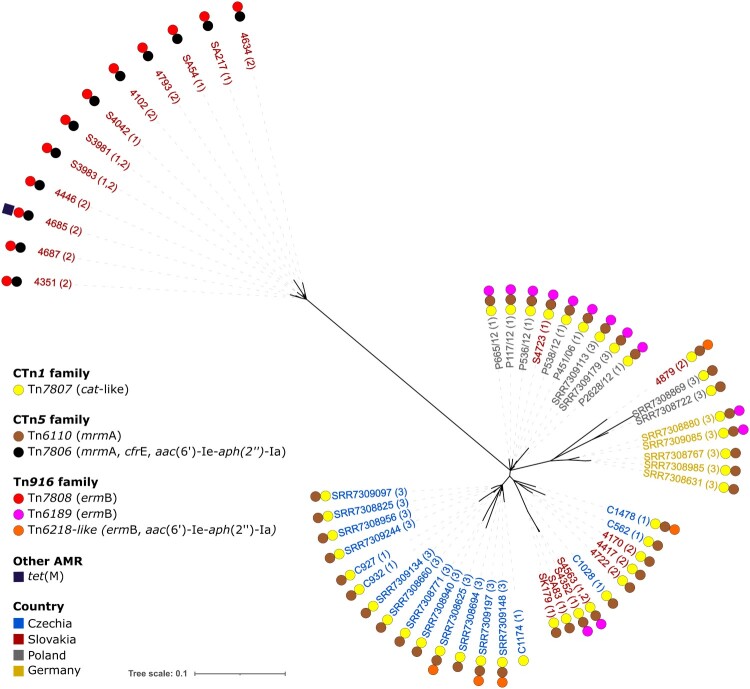
**Unrooted Maximum-Likelihood phylogenetic tree with additional RT176 *C. difficile* isolates.** The country of origin of *C. difficile* isolates is colour-coded as shown in legend and the study origin is depicted by numbers in brackets: (1) isolates from this study, (2) isolates from [10], (3) isolates from [34]. A square indicates the presence of the *tet*M gene, while circles represent the presence of integrative conjugative elements from each family.

Two putative integrative mobilizable elements without any cargo genes were also identified. A 5.5 kb element with 100% identity to IME_CdiR20291_ND from *C. difficile* R20291 [44] and a 5.1 kb putative transposon which is present only in the Polish isolates and Slovak isolate (S4723). Additionally, several phage-like elements are present in all sequenced genomes and include a prophage identical to Genbank accession number PP767789.1, a bacteriocin gene cluster [45] and a *skin*^Cd^ element disrupting the *sig*K sporulation gene [46]. Though isolate C562 and S4723 have lost parts of *skin*^Cd^, the *van*Z1 gene associated with low-level teicoplanin resistance [47] is preserved. However, we could not confirm a resistance phenotype in our susceptibility testing. The integrative and conjugative transposons were also searched in the full set of 22 *C. difficile* genomes from this study and in the extended set of 54 genomes from studies [10, 34]. The transposons Tn*6218* and Tn*6189*-like were distributed unevenly in several lineages in different variants and integration sites (Figure 4). Also, strain 4685 harboured one more integrative mobilizable element with *tet*M (Figure 5) and exhibited resistance to tetracycline (16 mg/L).

In summary, *C. difficile* PCR ribotype 176 isolates carry already described and unique transposons with antimicrobial resistance and virulence genes. The isolates formed four genetically related clusters and one distinct cluster with the co-presence of Tn*7806* and Tn*7808* in only Slovak isolates.

Remarkably, Tn*6105* encodes a previously uncharacterized 23S rRNA methyltransferase (WP_002584956.1), which is classified as a ribosomal RNA methyltransferase of the large subunit RlmN/Cfr (IPR004383) (Figure 2a and c) but forms a phylogenetic lineage distinct from the RlmN and Cfr [37]. Cfr methylates the C-8 of the 23S rRNA nucleotide A2503 [48] and confers resistance to phenicol, lincosamide, oxazolidinone, pleuromutilin, and streptogramin A antimicrobial classes [49]. In contrast, RlmN is a housekeeping methyltransferase that modifies C-2 of the same nucleotide [50]. All 21 *C. difficile* isolates containing Tn*6110* and Tn*7806* carry a chromosomal *rlm*N gene, which likely encodes a housekeeping enzyme (Figure 2c). Despite lacking the canonical *erm*B gene, all six isolates harbouring the 23S rRNA methyltransferase on Tn*6110* or Tn*7806* exhibited high-level erythromycin resistance (>256 mg/L) (Figure 4). These findings indicate a potentially novel role for this RlmN-like protein in mediating macrolide resistance.

To substantiate this hypothesis, we functionally characterized the identified *rlm*N-like gene. The gene was cloned into an L-arabinose inducible expression vector (pBAD30) and transformed into *E. coli* strain BW25113 Δ*tolC* (JW5503-1) that is susceptible to macrolides [39]. Importantly, previous studies have demonstrated that *cfr*-mediated resistance in *E. coli* necessitates pre-induction of gene expression before antimicrobial exposure [38]. Consequently, we pre-induced gene expression by culturing the transformed strain on L-arabinose-containing agar before antimicrobial susceptibility testing. The expression of his-tagged MrmA protein was confirmed by small-scale metal affinity chromatography followed by mass spectrometry (Figure S3).

To investigate the effects of the RlmN-like gene on antimicrobial susceptibility, we compared MICs of isolates containing the *rlmN*-like gene plasmid p6hisMrmA with a control vector (Table 1). We observed a dramatic 512-fold increase in the MIC of erythromycin and a modest increase in the MIC for streptogramin B (4-fold) and the combination of streptogramin A and B (3-fold). On the other hand, the MICs against the 16-membered macrolide tylosin, the streptogramin A antimicrobial pristinamycin IIA, the lincosamide clindamycin, the pleuromutilin antimicrobial tiamulin or against linezolid and chloramphenicol (oxazolidinones and phenicols, respectively) remained unchanged. These data identify the RlmN-like protein as a novel determinant of resistance specifically to 14-membered macrolides and streptogramins B. We propose the name *mrm*A (macrolide resistance methyltransferase A) for this gene.

**Table 1.** The minimum inhibitory concentrations of *E. coli* strains containing the RlmN-like gene plasmid p6hisMrmA with a control vector.

*E. coli* strains	*mrmA*	MIC (µg/mL)								
		Erythromycin	Tylosin	Pristinamycin IA	Pristinamycin IIA	Quinupristin Dalfopristin	Clindamycin	Tiamulin	Chloramphenicol	Linezolid
*ΔtolC*	-	2	16	128	4	1.5-2	4	0.25	2	2
*ΔtolC* + pBAD30	-	2	16	64–128	4	1.5-2	8	0.25	2	2
*ΔtolC* + p6hisMrmA	+	512–1024	16	1024	2	6	4	0.125-0.25	1	2

In addition to resistance, Tn*6110* and Tn*7806* also carried the putative virulence gene for the virulence-associated protein E-like with unknown function (98.2% similarity to *Erysipelotrichaceae* bacterium 5_2_54FAA) and genes encoding the VirB1-like, VirB4, VirB6 components of the type IV secretory system (T4SS type 4C). The gene encoding the fourth component of this T4SS type, VirD4, was disrupted by the insertion of Tn*6105* into Tn*6110* and by the additional insertion of the *cfr*E fragment in the case of Tn*7806* (Figure 2a, Supplementary data Table S4). Interestingly, the amino acid alignments of the hybrid VirB protein with the closest protein homologues showed an in-frame fusion of two *vir*D4 genes after the insertion of the *cfr*E gene-carrying fragment in Tn*6110*, possibly leading to an active protein (Figure S4).

## Discussion

Since 2003, the epidemiology of CDI has changed with the predominance of certain types with differences in individual European countries and regions [2]. *C. difficile* RT176 is frequently identified in Eastern Europe, [2] but with noticeable differences in time and between individual countries [6–8,10]. We investigated the genomic properties of *C. difficile* RT176 isolates from three countries. Since 7-locus MLVA has been reported to provide similar discriminatory power as WGS with single nucleotide variants (SNVs), irrespective of the PCR ribotype [51] we used MLVA for the selection of isolates for further investigation.

The MLVA identified six intercountry clonal complexes (CC 1-6) in 509 *C. difficile* RT176 isolates but cgMLST analysis of 22 sequenced clonally related pairs of isolates showed allele differences between 10–37 which do not meet the proposed adjusted threshold of zero to three allelic differences for outbreak recognition [4]. Our observation is not in the line of a 95% concordance between MLVA and WGS published by Eyre et al. [51], however, this concordance was not tested on a collection of RT176 isolates. We, therefore, conclude that MLVA has less discriminatory power than cgMLST for RT176.

The genetic relatedness of RTs 176 and 027 has been already described [1,3,4] and isolates in our study also shared notoriously known molecular patterns as they belonged to clade 2 and sequence type 1, carried genes for binary toxin, and had an 18 bp deletion at position 311 and a single nucleotide deletion at position 117 in the *tcdC* gene.

From antimicrobials tested, all RT176 isolates were resistant to fluoroquinolones (ciprofloxacin and moxifloxacin) and carried mutations resulting in an amino acid substitution T82I in GyrA protein, that is also present in epidemic lineages belonging to RT027 [1] and to amikacin and gentamicin with *aac*(6’)-*Ie-aph*(2'’)-*Ia* gene present in 6/22 isolates. *C. difficile* is inherently resistant to aminoglycosides [52], so the reason for the acquisition of *aac*(6’)-*Ie-aph*(2'’)-*Ia* genes is unclear, but the geometric mean MIC of gentamicin and amikacin in our study was higher in isolates that carried the resistance determinant (mean MICs >256 mg/L and >256 mg/L vs to 55.4 and 117.4 mg/L, respectively). Furthermore, we identified five isolates with resistance to imipenem (≥32 mg/L) and carrying mutations resulting in P491L substitutions in PBP1 and N537K substitutions in PBP3. These substitutions were previously identified in 120 genomes of 10 STs and 7 genomes of ST1 [53] but unfortunately, the relation between the genotype and phenotype was not investigated. We note that imipenem susceptible isolates in our study did not have the above-mentioned genotype, suggesting a possible causal relationship of these mutations with the resistance phenotype.

An important discrepancy was found between predicted genotype resistance and detected phenotype resistance for *cfr*E (predicted, lincosamides and linezolid resistance), *van*Z1 (predicted teicoplanin resistance), *cat-*like (predicted chloramphenicol resistance) genes and the P*nimB*^G^ mutation (predicted metronidazole resistance). For *cfr*E, this might be related to rearrangements in the *cfr*E upstream region as previously described [10]. An effect on clindamycin resistance was not observed because the isolates also carried the *erm*B gene. We found the presence of *van*Z1 not to be associated with low teicoplanin resistance, unlike others [46]. The *cat*-like gene detected in our isolates showed 36% similarity to the *cat* gene from *C. perfringens* but also for this gene, the susceptibility data do not support its chloramphenicol-resistance function in *C. difficile*. In the extended dataset, only strain S4685 carried the *tet*M gene located on a small insert.

A T-to-G mutation in the promoter of the *nim*B gene was recently described to be associated with heme-dependent resistance to metronidazole [27]. Though all isolates were found to be susceptible to metronidazole on EUCAST-recommended media, we observed that when isolates were retested on hemin-enriched media (chocolate agar), 5/22 showed MICs between 3 or 4 mg/L which would qualify them as resistant. In our isolates, a mutation resulting in an additional amino acid substitution (L155I) in NimB was present; the effect of this mutation on the metronidazole resistance phenotype has not been determined. Therefore, further investigation into the genetic determinants of heme-dependent metronidazole resistance of RT176 is needed.

Resistance to both erythromycin and clindamycin in the investigated RT176 isolates was explained by the presence of the *erm*B gene, which was surprisingly carried by three different transposons of Tn*916* family. Tn*7808* which was present in one separate lineage of Slovak isolates and Tn*6218* and Tn*6189* which are distributed unevenly between several lineages and integrated in multiple chromosomal sites (Figure 5) suggesting independent horizontal transmission events. We observed a high level of resistance to erythromycin with a concomitant susceptibility to clindamycin in six *C. difficile* isolates that were not explained by the presence of known resistance determinants or by mutations in 23S rRNA and has also been observed in *C. difficile* isolates of different ribotypes [54,55]. Using a comparative genomics approach, we identified the novel resistance determinant *mrm*A carried by the transposons Tn*6110* and Tn*7806*. Tn*6110* was previously predicted to be important for macrolide resistance [56], but the responsible resistance determinant was not discovered. Here, we demonstrate that heterologous expression of the *mrm*A gene in *E. coli* confers resistance to erythromycin and streptogramin B, but not other ribosome-targeting antimicrobials. Although the inactivation of *mrm*A in *C. difficile* has not yet been directly linked to increased erythromycin susceptibility, the ability of *mrm*A to mediate resistance in a heterologous system and the strong correlation between *mrm*A presence and high-level erythromycin resistance in *erm*B-negative isolates strongly suggest a causative role.

MrmA belongs to the family of radical SAM-dependent methyltransferases, similar to Cfr and RlmN. However, Cfr and RlmN methylate distinct atoms within the same adenine residue (A2503 in *E. coli* numbering) of the 23S rRNA, and these modifications do not affect erythromycin activity [49]. We hypothesize that MrmA methylates a different adenine residue on the 23S rRNA compared to Cfr and RlmN, thereby specifically impacting erythromycin binding without affecting oxazolidinones, phenicols, pleuromutilins, lincosamides and 16-membered macrolides, to which *E. coli* strain expressing *mrm*A remained susceptible. This hypothesis is further supported by the lower sequence identity between MrmA and Cfr/RlmN compared to each other (Figure S5), suggesting a potentially unique substrate specificity for MrmA. Future studies are warranted to elucidate the precise methylation target(s) of MrmA.

Genome comparison identified six large inserts with distinct distributions. The presence of Tn*7806* together with Tn*7808* appears to be characteristic of the strains isolated recently in Slovakia, where *C. difficile* RT176 dominates (Figure 4, Figure 5). The same pattern was also found in 7 sequenced Slovak *C. difficile* RT176 isolates from the study by Plankaova et al. [10] which were selected randomly in one isolate per hospital. The frequency of individual inserts and their role in CDI epidemiology needs to be investigated in further studies. Apart from the *lsa*-like and *cfr*E resistance genes, which show no apparent linezolid resistance phenotype, Tn*7806* does not contain any genes that might favour the carrier strain except for the putative VirD4 component of the type 4C secretion system (T4CSS). T4CSS is the four-member secretory system, which was originally identified in *Streptococcus suis* [57,58] and partially characterized in *C. difficile* 630 [59]. T4CSS increases bacterial pathogenicity and could cause large-scale outbreaks of streptococcal infections in humans [58]. The Tn*6110* encodes VirB1-like, VirB4, VirB6 and VirD4 components of the type IV secretory system in which the *virD4* was disrupted by the insertion of Tn*6105*. However, the additional insertion of a *cfr*E-carrying insert that contains another *vir*D4-like gene resulted in the in-frame fusion of corresponding parts of *vir*D genes that might result in a functional VirD4 hybrid (Figure 2b, S3). Therefore, the insertion of the *cfr*E fragment can lead to the functionality of the entire T4CSS being restored.

## Conclusion

Multidrug-resistant *C. difficile* RT176 isolates carry already described and unique transposons with antimicrobial resistance and virulence genes and formed four genetically related and one distinct cluster with the co-presence of Tn*7806* and Tn*7808* in only Slovak isolates. The novel, RlmN type 23S rRNA methyltransferase, designated MrmA, that correlates with the high level of erythromycin resistance in isolates without *erm*B was identified.

## Supplementary Material

Table_S1_2_3.docx

Table S4.xlsx

FigS5.jpg

FigS1.jpg

FigS4.jpg

FigS2.jpg

FigS3.tiff

## Data access

All raw sequence data and annotated hybrid assemblies of *C. difficile* isolates C562, C1174, C1478, S3981 and S4723 from this study were submitted to the NCBI Sequence Read Archive (SRA) under accession number BioProject ID PRJNA1081163 (more details in Table S3).

## References

[1] He M, Miyajima F, Roberts P, et al. Emergence and global spread of epidemic healthcare-associated *Clostridium difficile*. Nat Genet. 2013;45(1):109–113. doi:10.1038/ng.2478 23222960 PMC3605770

[2] Viprey VF, Davis GL, Benson AD, et al. A point-prevalence study on community and inpatient *Clostridioides difficile* infections (CDI): results from combatting bacterial resistance in Europe CDI (COMBACTE-CDI), July to November 2018. Euro Surveill. 2022;27(26):2100704, doi:10.2807/1560-7917.ES.2022.27.26.2100704 35775426 PMC9248264

[3] Krutova M, Wilcox MH, Kuijper EJ. The pitfalls of laboratory diagnostics of *Clostridium difficile* infection. Clin Microbiol Infect. 2018;24(7):682–683. doi:10.1016/j.cmi.2018.02.026 29505883

[4] Baktash A, Corver J, Harmanus C, et al. Comparison of whole-genome sequence-based methods and PCR ribotyping for subtyping of *Clostridioides difficile*. J Clin Microbiol. 2022;60(2):e0173721, doi:10.1128/JCM.01737-21 34911367 PMC8849210

[5] European Centre for Disease Prevention and Control. Study protocol for a survey of whole genome sequencing of *Clostridioides difficile* isolates from tertiary acute care hospitals, EU/EEA, 2022–2023. Stockholm: ECDC; 2024.

[6] Pituch H, Obuch-Woszczatyński P, Lachowicz D, et al. Hospital-based *Clostridium difficile* infection surveillance reveals high proportions of PCR ribotypes 027 and 176 in different areas of Poland, 2011 to 2013. Euro Surveill. 2015;20(38), doi:10.2807/1560-7917.ES.2015.20.38.30025 26536049

[7] Krutova M, Nyc O, Kuijper EJ, et al. A case of imported *Clostridium difficile* PCR-ribotype 027 infection within the Czech Republic which has a high prevalence of *C. difficile* ribotype 176. Anaerobe. 2014;30:153–155. doi:10.1016/j.anaerobe.2014.09.020 25300750

[8] Krutova M, Capek V, Nycova E, et al. The association of a reduced susceptibility to moxifloxacin in causative *Clostridium* (*Clostridioides*) *difficile* strain with the clinical outcome of patients. Antimicrob Resist Infect Control. 2020;9(1):98, doi:10.1186/s13756-020-00765-y 32605598 PMC7325081

[9] Nyc O, Krutova M, Liskova A, et al. The emergence of *Clostridium difficile* PCR-ribotype 001 in Slovakia. Eur J Clin Microbiol Infect Dis. 2015;34(8):1701–1708. doi:10.1007/s10096-015-2407-9 25981433

[10] Plankaova A, Brajerova M, Capek V, et al. *Clostridioides difficile* infections were predominantly driven by fluoroquinolone-resistant *Clostridioides difficile* ribotypes 176 and 001 in Slovakia in 2018-2019. Int J Antimicrob Agents. 2023;62(1):106824, doi:10.1016/j.ijantimicag.2023.106824 37116667

[11] Novakova E, Kotlebova N, Gryndlerova A, et al. An outbreak of *Clostridium* (*Clostridioides*) *difficile* infections within an acute and long-term care wards due to moxifloxacin-resistant PCR ribotype 176 genotyped as PCR ribotype 027 by a commercial assay. J Clin Med. 2020;9(11):3738, doi:10.3390/jcm9113738 33233843 PMC7699857

[12] Krehelova M, Nyč O, Sinajová E, et al. The predominance and clustering of *Clostridioides* (*Clostridium*) *difficile* PCR ribotype 001 isolates in three hospitals in eastern Slovakia, 2017. Folia Microbiol (Praha). 2019;64(1):49–54. doi:10.1007/s12223-018-0629-9 29971567

[13] Krutova M, Matejkova J, Kuijper EJ, et al. *Clostridium difficile* PCR ribotypes 001 and 176 - the common denominator of *C. difficile* infection epidemiology in the Czech Republic, 2014. Euro Surveill. 2016;21(29), doi:10.2807/1560-7917.ES.2016.21.29.30296 27484171

[14] Karpiński P, Wultańska D, Piotrowski M, et al. Motility and the genotype diversity of the flagellin genes *fliC* and *fliD* among *Clostridioides difficile* ribotypes. Anaerobe. 2022;73:102476, doi:10.1016/j.anaerobe.2021.102476 34780914

[15] Fawley WN, Knetsch CW, MacCannell DR, et al. Development and validation of an internationally-standardized, high-resolution capillary gel-based electrophoresis PCR-ribotyping protocol for *Clostridium difficile*. PLoS One. 2015;10(2):e0118150, doi:10.1371/journal.pone.0118150 25679978 PMC4332677

[16] Persson S, Torpdahl M, Olsen KE. New multiplex PCR method for the detection of *Clostridium difficile* toxin A (*tcdA*) and toxin B (*tcdB*) and the binary toxin (*cdtA*/*cdtB*) genes applied to a Danish strain collection. Clin Microbiol Infect. 2008;14(11):1057–1064. doi:10.1111/j.1469-0691.2008.02092.x. Erratum in: Clin Microbiol Infect. 2009;15(3):296. 19040478

[17] van den Berg RJ, Schaap I, Templeton KE, et al. Typing and subtyping of *Clostridium difficile* isolates by using multiple-locus variable-number tandem-repeat analysis. J Clin Microbiol. 2007;45(3):1024–1028. 17166961 10.1128/JCM.02023-06 PMC1829118

[18] Prjibelski A, Antipov D, Meleshko D, et al. Using SPAdes De novo assembler. Curr Protocols Bioinf. 2020;70(1), doi:10.1002/cpbi.102 32559359

[19] Kolmogorov M, Yuan J, Lin Y, et al. Assembly of long, error-prone reads using repeat graphs. Nat Biotechnol. 2019;37(5):540–546. doi:10.1038/s41587-019-0072-8 30936562

[20] Wick RR, Holt KE. Polypolish: short-read polishing of long-read bacterial genome assemblies. PLoS Comput Biol. 2022;18(1):e1009802, doi:10.1371/journal.pcbi.1009802 35073327 PMC8812927

[21] Kaas RS, Leekitcharoenphon P, Aarestrup FM, et al. Solving the problem of comparing whole bacterial genomes across different sequencing platforms. PLoS One. 2014;9(8):e104984, doi:10.1371/journal.pone.0104984 25110940 PMC4128722

[22] Kozlov AM, Darriba D, Flouri T, et al. RAxML-NG: a fast, scalable and user-friendly tool for maximum likelihood phylogenetic inference. Bioinformatics. 2019;35(21):4453–4455. doi:10.1093/bioinformatics/btz305 31070718 PMC6821337

[23] Letunic I, Bork P. Interactive Tree of Life (iTOL) v6: recent updates to the phylogenetic tree display and annotation tool. Nucleic Acids Res. 2024;52(W1):W78–W82. doi:10.1093/nar/gkae268 38613393 PMC11223838

[24] Torsten S. (2015). “Abricate.” Github. https://github.com/tseemann/abricate.

[25] Isidro J, Santos A, Nunes A, et al. Imipenem resistance in *Clostridium difficile* ribotype 017, Portugal. Emerg Infect Dis. 2018;24(4):741–745. doi:10.3201/eid2404 29553322 PMC5875251

[26] Boekhoud IM, Sidorov I, Nooij S, et al. Haem is crucial for medium-dependent metronidazole resistance in clinical isolates of *Clostridioides difficile*. J Antimicrob Chemother. 2021;76(7):1731–1740. doi:10.1093/jac/dkab097 33876817 PMC8212768

[27] Olaitan AO, Dureja C, Youngblom MA, et al. Decoding a cryptic mechanism of metronidazole resistance among globally disseminated fluoroquinolone-resistant *Clostridioides difficile*. Nat Commun. 2023;14(1):4130, doi:10.1038/s41467-023-39429-x 37438331 PMC10338468

[28] Smits WK, Harmanus C, Sanders IMJG, et al. Sequence-Based identification of metronidazole-resistant *Clostridioides difficile* isolates. Emerg Infect Dis. 2022;28(11):2308–2311. doi:10.3201/eid2811.220615 36286226 PMC9622256

[29] Sullivan MJ, Petty NK, Beatson SA. Easyfig: a genome comparison visualizer. Bioinformatics. 2011;27(7):1009–1010. doi:10.1093/bioinformatics/btr039 21278367 PMC3065679

[30] Bertelli C, Laird MR, Williams KP, et al. Islandviewer 4: expanded prediction of genomic islands for larger-scale datasets. Nucleic Acids Res. 2017;45(W1):W30–W35. doi:10.1093/nar/gkx343 28472413 PMC5570257

[31] Johansson MHK, Bortolaia V, Tansirichaiya S, et al. Detection of mobile genetic elements associated with antibiotic resistance in *Salmonella enterica* using a newly developed web tool: MobileElementFinder. J Antimicrob Chemother. 2021;76(1):101–109. doi:10.1093/jac/dkaa390 33009809 PMC7729385

[32] Wang M, Liu G, Liu M, et al. Iceberg 3.0: functional categorization and analysis of the integrative and conjugative elements in bacteria. Nucleic Acids Res. 2024;52(D1):D732–D737. doi:10.1093/nar/gkad935 37870467 PMC10767825

[33] Roberts AP, Chandler M, Courvalin P, et al. Revised nomenclature for transposable genetic elements. Plasmid. 2008;60(3):167–173. doi:10.1016/j.plasmid.2008.08.001 18778731 PMC3836210

[34] Eyre DW, Davies KA, Davis G, et al. Two Distinct Patterns of Clostridium *difficile* Diversity Across Europe Indicating Contrasting Routes of Spread. Clin Infect Dis. 2018;67(7):1035–1044. doi:10.1093/cid/ciy252 29659747 PMC6137122

[35] Cizek A, Masarikova M, Mares J, et al. Detection of plasmid-mediated resistance to metronidazole in *Clostridioides difficile* from river water. Microbiol Spectr. 2022;10(4):e0080622, doi:10.1128/spectrum.00806-22 35950844 PMC9431275

[36] Yan F, LaMarre JM, Röhrich R, et al. Rlmn and Cfr are radical SAM enzymes involved in methylation of ribosomal RNA. J Am Chem Soc. 2010;132(11):3953–3964. doi:10.1021/ja910850y 20184321 PMC2859901

[37] Atkinson GC, Hansen LH, Tenson T, et al. Distinction between the Cfr methyltransferase conferring antibiotic resistance and the housekeeping RlmN methyltransferase. Antimicrob Agents Chemother. 2013;57(8):4019–4026. doi:10.1128/AAC.00448-13 23752511 PMC3719738

[38] Hansen LH, Vester B. A *cfr*-like gene from *Clostridium difficile* confers multiple antibiotic resistance by the same mechanism as the *cfr* gene. Antimicrob Agents Chemother. 2015;59(9):5841–5843. doi:10.1128/AAC.01274-15 26149991 PMC4538495

[39] Baba T, Ara T, Hasegawa M, et al. Construction of *Escherichia coli* K-12 in-frame, single-gene knockout mutants: the Keio collection. Mol Syst Biol. 2006;2; doi:10.1038/msb4100050 PMC1681482 16738554

[40] Sebaihia M, Wren BW, Mullany P, et al. The multidrug-resistant human pathogen *Clostridium difficile* has a highly mobile, mosaic genome. Nat Genet. 2006;38(7):779–786. doi:10.1038/ng1830 16804543

[41] Brouwer MS, Warburton PJ, Roberts AP, et al. Genetic organisation, mobility and predicted functions of genes on integrated, mobile genetic elements in sequenced strains of *Clostridium difficile*. PLoS One. 2011;6(8):e23014, doi:10.1371/journal.pone.0023014 21876735 PMC3158075

[42] Singh KV, Weinstock GM, Murray BE. An *Enterococcus faecalis* ABC homologue (Lsa) is required for the resistance of this species to clindamycin and quinupristin-dalfopristin. Antimicrob Agents Chemother. 2002;46(6):1845–1850. doi:10.1128/AAC.46.6.1845-1850.2002 12019099 PMC127256

[43] Rice LB, Carias LL, Marshall S, et al. Tn*5386*, a novel Tn*916*-like mobile element in *Enterococcus faecium* D344R that interacts with Tn*916* to yield a large genomic deletion. J Bacteriol. 2005;187(19):6668–6677. doi:10.1128/JB.187.19.6668-6677.2005 16166528 PMC1251567

[44] Guédon G, Lao J, Payot S, et al. FirmiData: a set of 40 genomes of Firmicutes with a curated annotation of ICEs and IMEs. BMC Res Notes. 2022;15(1):157, doi:10.1186/s13104-022-06036-w 35538580 PMC9092696

[45] Gebhart D, Williams SR, Bishop-Lilly KA, et al. Novel high-molecular-weight, R-type bacteriocins of *Clostridium difficile*. J Bacteriol. 2012;194(22):6240–47. doi:10.1128/jb.01272-12. 22984261 PMC3486368

[46] Haraldsen JD, Sonenshein AL. Efficient sporulation in *Clostridium difficile* requires disruption of the σ^K^ gene. Molecular Microbiology. 2003;48(3):811–21. doi:10.1046/j.1365-2958.2003.03471.x. 12694623

[47] Woods EC, Wetzel D, Mukerjee M, et al. Examination of the *Clostridioides* (*Clostridium*) *difficile* VanZ ortholog, CD1240. Anaerobe. 2018;53:108–115. doi:10.1016/j.anaerobe.2018.06.013. 29940245 PMC6309587

[48] Giessing AM, Jensen SS, Rasmussen A, et al. Identification of 8-methyladenosine as the modification catalyzed by the radical SAM methyltransferase Cfr that confers antibiotic resistance in bacteria. RNA. 2009;15(2):327–336. doi:10.1261/rna.1371409 19144912 PMC2648713

[49] Long KS, Poehlsgaard J, Kehrenberg C, et al. The Cfr rRNA methyltransferase confers resistance to Phenicols, Lincosamides, Oxazolidinones, Pleuromutilins, and Streptogramin A antibiotics. Antimicrob Agents Chemother. 2006;50(7):2500–2505. doi:10.1128/AAC.00131-06 16801432 PMC1489768

[50] Toh SM, Xiong L, Bae T, et al. The methyltransferase YfgB/RlmN is responsible for modification of adenosine 2503 in 23S rRNA. RNA. 2008;14(1):98–106. doi:10.1261/rna.814408 18025251 PMC2151032

[51] Eyre DW, Fawley WN, Best EL, et al. Comparison of multilocus variable-number tandem-repeat analysis and whole-genome sequencing for investigation of *Clostridium difficile* transmission. J Clin Microbiol. 2013;51(12):4141–4149. doi:10.1128/JCM.01095-13 24108611 PMC3838059

[52] Khanafer N, Daneman N, Greene T, et al. Susceptibilities of clinical *Clostridium difficile* isolates to antimicrobials: a systematic review and meta-analysis of studies since 1970. Clin Microbiol Infect. 2018;24(2):110–117. doi:10.1016/j.cmi.2017.07.012 28750918

[53] Dingle KE, Freeman J, Didelot X, et al. Penicillin binding protein substitutions cooccur with fluoroquinolone resistance in epidemic lineages of multidrug-resistant *Clostridioides difficile*. mBio. 2023;14(2):e0024323, doi:10.1128/mbio.00243-23 37017518 PMC10128037

[54] Krutova M, Matejkova J, Tkadlec J, et al. Antibiotic profiling of *Clostridium difficile* ribotype 176–A multidrug resistant relative to *C. difficile* ribotype 027. Anaerobe. 2015;36:88–90. doi:10.1016/j.anaerobe.2015.07.009 26256807

[55] Spigaglia P, Barbanti F, Mastrantonio P, et al. Multidrug resistance in European *Clostridium difficile* clinical isolates. J Antimicrob Chemother. 2011;66(10):2227–2234. doi:10.1093/jac/dkr292 21771851

[56] Kaminska KH, Purta E, Hansen LH, et al. Insights into the structure, function and evolution of the radical-SAM 23S rRNA methyltransferase Cfr that confers antibiotic resistance in bacteria. Nucleic Acids Res. 2010;38(5):1652–1663. 20007606 10.1093/nar/gkp1142 PMC2836569

[57] Li M, Shen X, Yan J, et al. GI-type T4SS-mediated horizontal transfer of the 89 K pathogenicity island in epidemic *Streptococcus suis* serotype 2. Mol Microbiol. 2011;79(6):1670–1683. doi:10.1111/j.1365-2958.2011.07553.x 21244532 PMC3132442

[58] Zhang W, Rong C, Chen C, et al. Type-IVC secretion system: a novel subclass of type IV secretion system (T4SS) common existing in gram-positive genus *Streptococcus*. PLoS One. 2012;7(10):e46390, doi:10.1371/journal.pone.0046390 23056296 PMC3464263

[59] Sorokina J, Sokolova I, Rybolovlev I, et al. VirB4- and VirD4-like ATPases, components of a putative type 4C secretion system in *Clostridioides difficile*. J Bacteriol. 2021;203(21):e0035921, doi:10.1128/JB.00359-21 34424036 PMC8508108

