# Supplementary material for: Genomic islands and molecular mechanisms relating to drug-resistance in Clostridioides (Clostridium) difficile PCR ribotype 176
Source: Emerg Microbes Infect. 2025 Mar 25;14(1):2482698. doi: 10.1080/22221751.2025.2482698 (PMC11983580; doi:10.1080/22221751.2025.2482698)
Supplement: Table_S1_2_3.docx [file TEMI_A_2482698_SM1069.docx]

"This supplementary material is hosted by (name of the journal) as supporting information alongside the article "Genomic islands and molecular mechanisms relating to drug-resistance in *Clostridioides* (*Clostridium*) *difficile* PCR ribotype 176.", on behalf of the authors, who remain responsible for the accuracy and appropriateness of the content. The same standards for ethics, copyright, attributions and permissions as for the article apply. "

**Table S1: Characteristics of hybrid assemblies of five selected strains of *C. difficile***

| ***C. difficile* isolate ID** | **Genbank Accession** | **Contigs** | **CDS** | **CG (%)** | **Coverage** | **Length (bp)** |
| --- | --- | --- | --- | --- | --- | --- |
| C562 | CP160824 | contig_1 | 3754 | 30.95 | 256 | 4163377 |
| C1174 | CP160825 | contig_1 | 3716 | 30.91 | 32 | 4114413 |
| C1478 | JBFMIH000000000 | contig_2 | 3781 | 28.74 | 237 | 418866 |
|  |  | contig_1 | 4 | 43.38 | 4241 | 3534 |
| S3981 | CP160826 | contig_1 | 3784 | 28.81 | 50 | 4194228 |
| S4723 | JBFMII000000000 | contig_1 | 3766 | 30.94 | 168 | 4164606 |
|  |  | contig_2 | 25 | 29.60 | 123 | 23994 |

**Table S2a: A summary of antimicrobial susceptibility testing**

| **Antimicrobial susceptibility** | **Vancomycin** | **Tigecycline** | **Fidaxomicin** | **Amoxicillin** | **Tetracycline** | **Metronidazole** | | | **P*nim*B*^G^* and *nim*B_p.L155I** |
| --- | --- | --- | --- | --- | --- | --- | --- | --- | --- |
| **Culture media** | **SA** | **SA** | **WCA** | **SA** | **SA** | **SA** | **FAA** | **CA** |  |
| **E-test range/#agar dilution [mg/L]** | 0-256 | 0-256 | 0.0039-0.125# | 0-256 | 0-256 | 0-256 | | |  |
| **Breakpoin/ECOFF [mg/L] (a. EUCAST, b.CLSI)** | >2^a^ | NA | >0.5^a^ | ≥16^b^ | ≥16^b^ | >2^a^ | | |  |
| **Isolate ID** |  |  |  |  |  |  |  |  |  |
| C562* | 0.38 | 0.12 | 0.0078 | 0.75 | 0.094 | 1 | 0.38 | 1.5 | y |
| C1478* | 0.38 | 0.03 | 0.03125 | 1 | 0.064 | 0.75 | 0.38 | 1 | y |
| C927 | 0.25 | 0.06 | 0.0156 | 1 | 0.064 | 0.5 | 2 | 3 | y |
| C932 | 0.5 | 0.12 | 0.0156 | 0.75 | 0.047 | 1.5 | 1 | 4 | y |
| C1028 | 0.38 | 0.06 | ≤0.0039 | 1 | 0.064 | 0.5 | 0.5 | 3 | y |
| C1174* | 0.25 | 0.06 | ≤0.0039 | 0.75 | 0.047 | 1 | 0.38 | 1.5 | y |
| SA83 | 0.25 | 0.12 | 0.0156 | 0.75 | 0.032 | 0.75 | 0.19 | 1.5 | y |
| P2628/12 | 0.5 | 0.016 | 0.0078 | 0.75 | 0.032 | 1 | 0.25 | 1.5 | y |
| S4563 | 0.19 | 0.03 | 0.0078 | 0.5 | 0.032 | 0.19 | 0.5 | 2 | y |
| P117/12 | 0.38 | 0.03 | 0.0078 | 0.5 | 0.032 | 0.5 | 0.094 | 1.5 | y |
| S4352 | 0.25 | 0.06 | 0.0156 | 0.25 | 0.032 | 0.25 | 0.25 | 1.5 | y |
| SA54 | 0.38 | 0.06 | 0.0078 | 0.75 | 0.032 | 0.38 | 0.75 | 4 | y |
| SA217 | 0.25 | 0.016 | 0.0156 | 1 | 0.032 | 0.38 | 0.38 | 1.5 | y |
| P536/12 | 0.25 | 0.016 | 0.0078 | 0.75 | 0.032 | 0.75 | 0.19 | 1.5 | y |
| P538/12 | 0.25 | 0.06 | 0.0078 | 0.75 | 0.047 | 0.5 | 0.75 | 4 | y |
| SK179 | 0.25 | 0.06 | 0.0039 | 0.5 | 0.047 | 0.75 | 0.19 | 1.5 | y |
| P451/06 | 0.38 | 0.06 | 0.0078 | 0.75 | 0.047 | 0.75 | 0.38 | 1.5 | y |
| S4723* | 0.25 | 0.06 | 0.0156 | 0.5 | 0.047 | 0.38 | 0.125 | 1.5 | y |
| P665/12 | 0.38 | 0.06 | ≤0.0039 | 0.75 | 0.064 | 2 | 0.125 | 1 | y |
| S4042 | 0.38 | 0.06 | ≤0.0039 | 1.5 | 0.047 | 0.75 | 0.25 | 1.5 | y |
| S3981* | 0.19 | 0.03 | 0.0078 | 0.75 | 0.023 | 0.19 | 0.19 | 1.5 | y |
| S3983 | 0.19 | 0.06 | 0.0078 | 1.5 | 0.064 | 0.75 | 0.19 | 1.5 | y |

**Table S2b: A summary of antimicrobial susceptibility testing**

| **Antimicrobial susceptibility** | **Linezolid** | ***cfr*E** | **Ciprofloxacin** | **Moxifloxacin** | ***gyr*A_p.T82I** | **Erythromycin** | **Clindamycin** | ***mrm*A *(erythromycin)*** | ***erm*B *(erythromycin and clindamycin)*** |
| --- | --- | --- | --- | --- | --- | --- | --- | --- | --- |
| **Culture media** | **SA** |  | **SA** | **SA** |  | **SA** | **SA** |  |  |
| **E-test range/#agar dilution [mg/L]** | 0-256 |  | 0-32 | 0-32 |  | 0-256 | 0-256 |  |  |
| **Breakpoin/ECOFF [mg/L] (a. EUCAST, b.CLSI)** | ≥4^b^ |  | ≥8^b^ | ≥8^b^ |  | ≥8^b^ | ≥8^b^ |  |  |
| **Isolate ID** |  |  |  |  |  |  |  |  |  |
| C562* | 2 | n | ≥32 | ≥32 | y | ≥256 | 2 | y | n |
| C1478* | 1.5 | n | ≥32 | ≥32 | y | ≥256 | ≥256 | y | y |
| C927 | 1.5 | n | ≥32 | ≥32 | y | ≥256 | 2 | y | n |
| C932 | 1 | n | ≥32 | ≥32 | y | ≥256 | 0.75 | y | n |
| C1028 | 1.5 | n | ≥32 | ≥32 | y | ≥256 | 2 | y | n |
| C1174* | 0.75 | n | ≥32 | ≥32 | y | 0.38 | 2 | n | n |
| SA83 | 0.75 | n | ≥32 | ≥32 | y | ≥256 | 2 | y | n |
| P2628/12 | 2 | n | ≥32 | ≥32 | y | ≥256 | ≥256 | y | y |
| S4563 | 0.38 | n | ≥32 | ≥32 | y | ≥256 | ≥256 | y | y |
| P117/12 | 2 | n | ≥32 | ≥32 | y | ≥256 | ≥256 | y | y |
| S4352 | 0.5 | n | ≥32 | ≥32 | y | ≥256 | ≥256 | y | y |
| SA54 | 1 | y | ≥32 | ≥32 | y | ≥256 | ≥256 | y | y |
| SA217 | 0.75 | y | ≥32 | ≥32 | y | ≥256 | ≥256 | y | y |
| P536/12 | 1 | n | ≥32 | ≥32 | y | ≥256 | ≥256 | y | y |
| P538/12 | 0.75 | n | ≥32 | ≥32 | y | ≥256 | ≥256 | y | y |
| SK179 | 1 | n | ≥32 | ≥32 | y | ≥256 | 2 | y | n |
| P451/06 | 1 | n | ≥32 | ≥32 | y | ≥256 | ≥256 | y | y |
| S4723* | 0.75 | n | ≥32 | ≥32 | y | ≥256 | ≥256 | y | y |
| P665/12 | 1 | n | ≥32 | ≥32 | y | ≥256 | ≥256 | y | y |
| S4042 | 1.5 | y | ≥32 | ≥32 | y | ≥256 | ≥256 | y | y |
| S3981* | 0.5 | y | ≥32 | ≥32 | y | ≥256 | ≥256 | y | y |
| S3983 | 1.5 | y | ≥32 | ≥32 | y | ≥256 | ≥256 | y | y |

**Table S2c: A summary of antimicrobial susceptibility testing**

| **Antimicrobial susceptibility** | **Gentamicin** | **Amikacin** | ***aac(6')-Ie-aph(2'')-Ia*** | **Rifampicin** | ***rpo*B** | **Imipenem** | ***pbp*1_P491L, *pbp*3_N537K** | **Ertapenem** | **Meropenem** | **Teicoplanin** | ***vanZ1*** | **Chloramphenicol** | ***cat*** |
| --- | --- | --- | --- | --- | --- | --- | --- | --- | --- | --- | --- | --- | --- |
| **Culture media** | **SA** | **SA** |  | **SA** |  | **SA** |  | **SA** | **SA** | **SA** |  | **SA** |  |
| **E-test range/#agar dilution [mg/L]** | 0-256 | 0-256 |  | 0-256 |  | 0-32 |  | 0-32 | 0-32 | 0-256 |  | 0-256 |  |
| **Breakpoin/ECOFF [mg/L] (a. EUCAST, b.CLSI)** | NA | NA |  | NA |  | ≥16^b^ |  | ≥16^b^ | ≥16^b^ | NA |  | NA |  |
| **Isolate ID** |  |  |  |  |  |  |  |  |  |  |  |  |  |
| C562* | 64 | ≥256 | n | ≥256 | R505K, D492E | 8 | n | 3 | 1.5 | 0.094 | y | 4 | y |
| C1478* | ≥256 | ≥256 | y | 0.016 | n | 8 | n | 6 | 2 | 0.125 | y | 4 | y |
| C927 | 96 | ≥256 | n | ≥256 | R505K, H502N | 12 | n | 6 | 2 | 0.19 | y | 8 | y |
| C932 | 48 | 64 | n | ≥256 | R505K, H502N | 8 | n | 3 | 1.5 | 0.19 | y | 6 | y |
| C1028 | 96 | ≥256 | n | ≥256 | R505K | 12 | n | 6 | 2 | 0.19 | y | 4 | y |
| C1174* | 48 | 64 | n | ≥256 | R505K, H502N | 8 | n | 4 | 1.5 | 0.094 | y | 4 | y |
| SA83 | 32 | 128 | n | ≥256 | R505K, H502N | 6 | n | 3 | 1.5 | 0.125 | y | 6 | y |
| P2628/12 | 32 | 64 | n | 0.016 | n | 8 | n | 4 | 1.5 | 0.064 | y | 6 | y |
| S4563 | 32 | 128 | n | ≥256 | R505K, H502N | 6 | n | 2 | 1 | 0.094 | y | 6 | y |
| P117/12 | 48 | 64 | n | 0.016 | n | 6 | n | 4 | 1.5 | 0.064 | y | 4 | y |
| S4352 | 24 | 64 | n | ≥256 | R505K, H502N | 6 | n | 2 | 0.75 | 0.125 | y | 3 | y |
| SA54 | ≥256 | ≥256 | y | 48 | R505K | ≥32 | y | 4 | 2 | 0.19 | y | 16 | n |
| SA217 | ≥256 | ≥256 | y | 64 | R505K | ≥32 | y | 12 | 4 | 0.125 | y | 6 | n |
| P536/12 | 64 | 32 | n | 0.016 | n | 12 | n | 8 | 2 | 0.125 | y | 4 | y |
| P538/12 | 64 | ≥256 | n | 0.016 | n | 6 | n | 2 | 1 | 0.125 | y | 4 | y |
| SK179 | 48 | 128 | n | ≥256 | R505K, H502N | 8 | n | 4 | 1.5 | 0.125 | y | 3 | y |
| P451/06 | 128 | 128 | n | 0.016 | n | 6 | n | 4 | 1.5 | 0.094 | y | 6 | y |
| S4723* | 48 | ≥256 | n | 0.016 | n | 8 | n | 4 | 1 | 0.094 | y | 4 | y |
| P665/12 | 128 | 128 | n | 0.016 | n | 8 | n | 6 | 2 | 0.094 | y | 4 | y |
| S4042 | ≥256 | ≥256 | y | ≥256 | R505K | ≥32 | y | 8 | 3 | 0.125 | y | 6 | n |
| S3981* | ≥256 | ≥256 | y | 64 | R505K | ≥32 | y | 6 | 2 | 0.094 | y | 6 | n |
| S3983 | ≥256 | ≥256 | y | 64 | R505K | ≥32 | y | 12 | 2 | 0.064 | y | 8 | n |

| A summary of antimicrobial susceptibility testing. Molecular determinants associated with particular resistance phenotype are at the same color. |
| --- |
| * *C. difficile* isolates with complete genome (hybrid assembly) |
| #agar dilution [mg/L] |
| y-yes. n-no |
| a) The European Committee on Antimicrobial Susceptibility Testing (EUCAST), epidemiological cut-off values (ECOFFs) |
| b) Clinical and Laboratory Standards Institute (CLSI) |
| NA: no breakpoints or ECOFFs for *C. difficile* were available |
| WCA - Wilkins Chalgren anaerobic agar |
| FAA - Fastidious anaerobe agar with horse blood |
| CA - Chocolate agar |
| SA - Schaedler agar |

**Table S2d:** **A summary of antimicrobial susceptibility testing**

| **Antimicrobials (E-test, *agar dilution)** | **MIC range [mg/L]** | **Culture media** | **Breakpoint/ECOFF value [mg/L]** | **Resistant (%)** | **Resistance determinant** |
| --- | --- | --- | --- | --- | --- |
| Vancomycin | 0.19-0.5 | SA | >2^a^ | none | - |
| Metronidazole | 0.19-2 | SA | >2^a^ | none | P*nim*B^G^, *nim*B_p.L155I |
|  | 0.094-2 | FAA |  | none |  |
|  | 1-4 | CA |  | 5 (22.7) |  |
| Fidaxomicin | ≤0.0039-0.03125 | WCA | >0.5^a^ | none | - |
| Tigecycline | 0.016-0.12 | SA | NA | - | - |
| Amoxicillin | 0.25-1.5 | SA | ≥16^b^ | - | - |
| Tetracycline | 0.023-0.094 | SA | ≥16^b^ | - | - |
| Linezolid | 0.38-2 | SA | ≥4^b^ | none | *cfr*E |
| Ciprofloxacin | >32 | SA | ≥8^b^ | 22 (100) | *gyr*A_p.T82I |
| Moxifloxacin |  |  |  |  |  |
| Erythromycin | 0.38->256 | SA | ≥8^b^ | 21 (95.5) | *mrm*A |
| Clindamycin | 0.75->256 |  |  | 15 (68.2) | *erm*B |
| Gentamicin | 24->256 | SA | NA | - | *aac*(6')-*Ie-aph*(2'')*-Ia* |
| Amikacin | 32->256 |  |  | - |  |
| Rifampicin | 0.016->256 | SA | NA | 14 (63.6) | *rpo*B_p. R505K, H502N, D492E |
| Imipenem | 6->32 | SA | ≥16^b^ | none | *pbp*1_P491L, *pbp*3_N537K |
| Ertapenem | 2-12 | SA | ≥16^b^ | none | - |
| Meropenem | 0.75-4 | SA | ≥16^b^ | none | - |
| Teicoplanin | 0.064-0.19 | SA | NA | none | *van*Z1 |
| Chloramphenicol | 3-16 | SA | NA | none | *cat* |

| a) The European Committee on Antimicrobial Susceptibility Testing (EUCAST), epidemiological cut-off values (ECOFFs) |
| --- |
| b) Clinical and Laboratory Standards Institute (CLSI) |
| NA: no breakpoints or ECOFFs for *C. difficile* were available |
| WCA - Wilkins Chalgren anaerobic agar |
| FAA - Fastidious anaerobe agar with horse blood |
| CA - Chocolate agar |
| SA - Schaedler agar |

Table S3: A Summary of molecular characterisation of C. difficile ribotype 176 isolates in the study and the sequence accession numbers.

| **Isolate ID** | **BioSample Accession number** | **Ribotype** | **ST** | **cgMLST Finder** | **Country** | **Year of culture** |
| --- | --- | --- | --- | --- | --- | --- |
| C562* | SAMN40150290 | 176 | 1 | 301 | CZ | 2014 |
| C1478* | SAMN40150291 | 176 | 1 | 4842 | CZ | 2014 |
| C927 | SAMN40150292 | 176 | 1 | 6593 | CZ | 2014 |
| C932 | SAMN40150293 | 176 | 1 | 6493 | CZ | 2014 |
| C1028 | SAMN40150294 | 176 | 1 | 301 | CZ | 2014 |
| C1174* | SAMN40150295 | 176 | 1 | 6593 | CZ | 2014 |
| SA83 | SAMN40150296 | 176 | 1 | 301 | SK | 2016 |
| P2628/12 | SAMN40150297 | 176 | 1 | 301 | PL | 2012 |
| S4563 | SAMN40150298 | 176 | 1 | 301 | SK | 2019 |
| P117/12 | SAMN40150299 | 176 | 1 | 301 | PL | 2012 |
| S4352 | SAMN40150300 | 176 | 1 | 301 | SK | 2019 |
| SA54 | SAMN40150301 | 176 | 1 | 301 | SK | 2016 |
| SA217 | SAMN40150302 | 176 | 1 | 301 | SK | 2016 |
| P536/12 | SAMN40150303 | 176 | 1 | 301 | PL | 2012 |
| P538/12 | SAMN40150304 | 176 | 1 | 301 | PL | 2012 |
| SK179 | SAMN40150305 | 176 | 1 | 301 | SK | 2017 |
| P451/06 | SAMN40150306 | 176 | 1 | 301 | PL | 2006 |
| S4723* | SAMN40150307 | 176 | 1 | 301 | SK | 2019 |
| P665/12 | SAMN40150308 | 176 | 1 | 301 | PL | 2012 |
| S4042 | SAMN40150309 | 176 | 1 | 301 | SK | 2018 |
| S3981* | SAMN40150310 | 176 | 1 | 301 | SK | 2018 |
| S3983 | SAMN40150311 | 176 | 1 | 301 | SK | 2018 |
