# Supplementary figures and images for: Genomic islands and molecular mechanisms relating to drug-resistance in Clostridioides (Clostridium) difficile PCR ribotype 176
Source: Emerg Microbes Infect. 2025 Mar 25;14(1):2482698. doi: 10.1080/22221751.2025.2482698 (PMC11983580; doi:10.1080/22221751.2025.2482698)

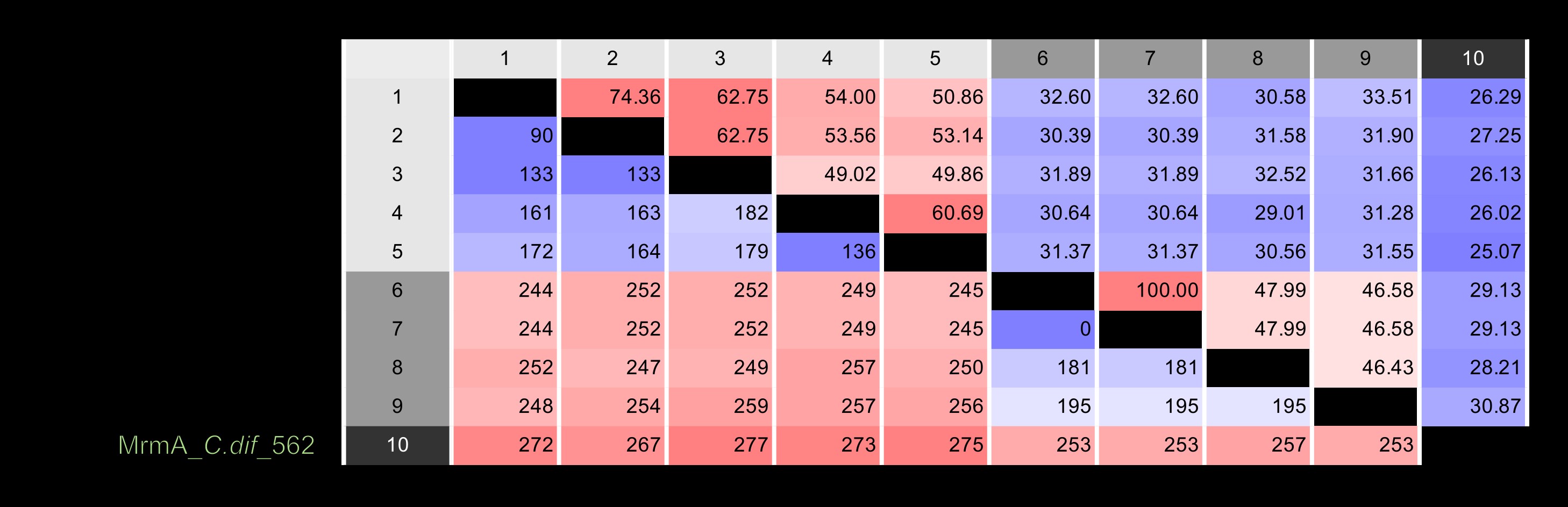

Supplement: FigS5.jpg [file TEMI_A_2482698_SM1067.jpg]

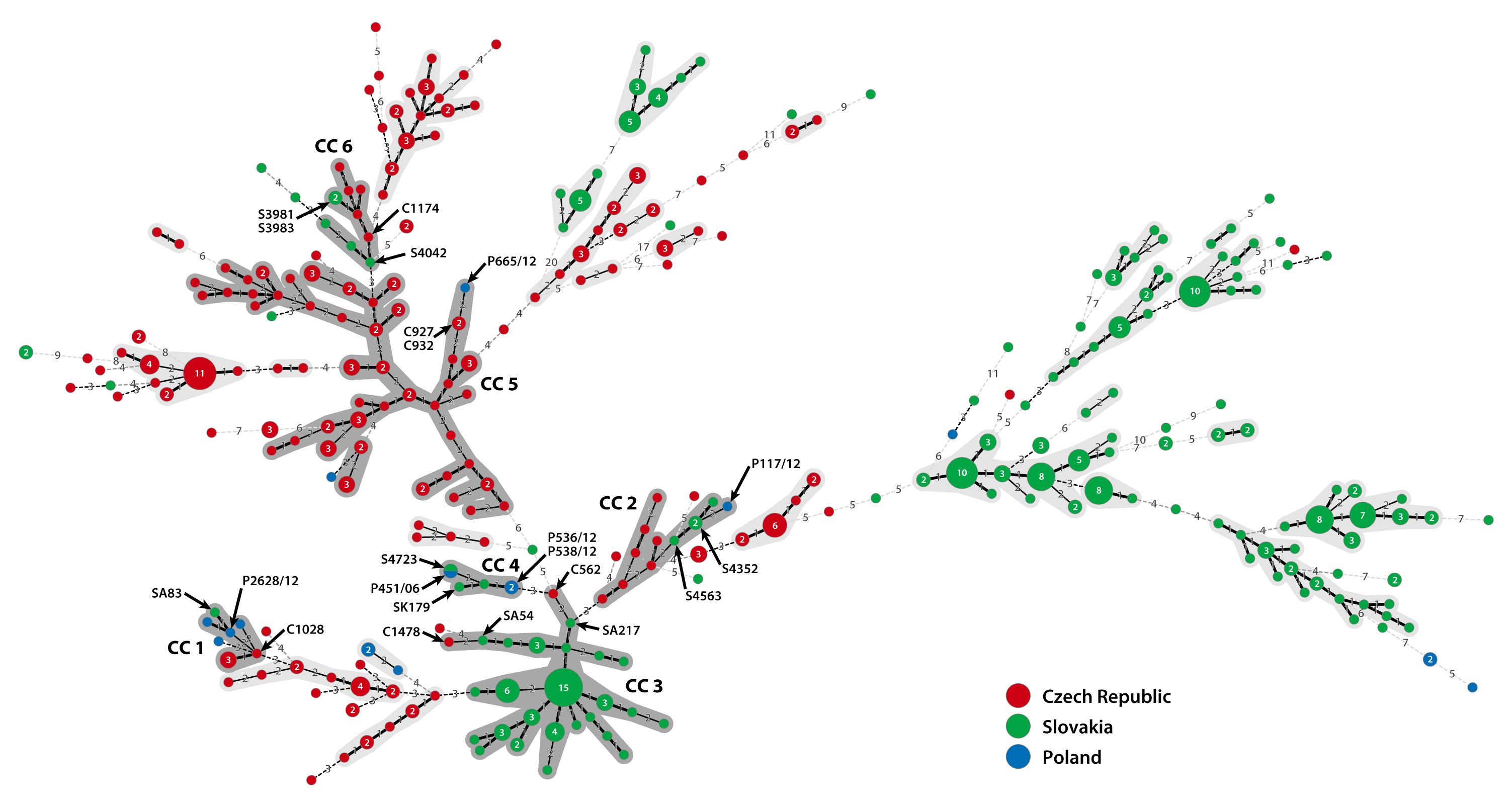

Supplement: FigS1.jpg [file TEMI_A_2482698_SM1066.jpg]

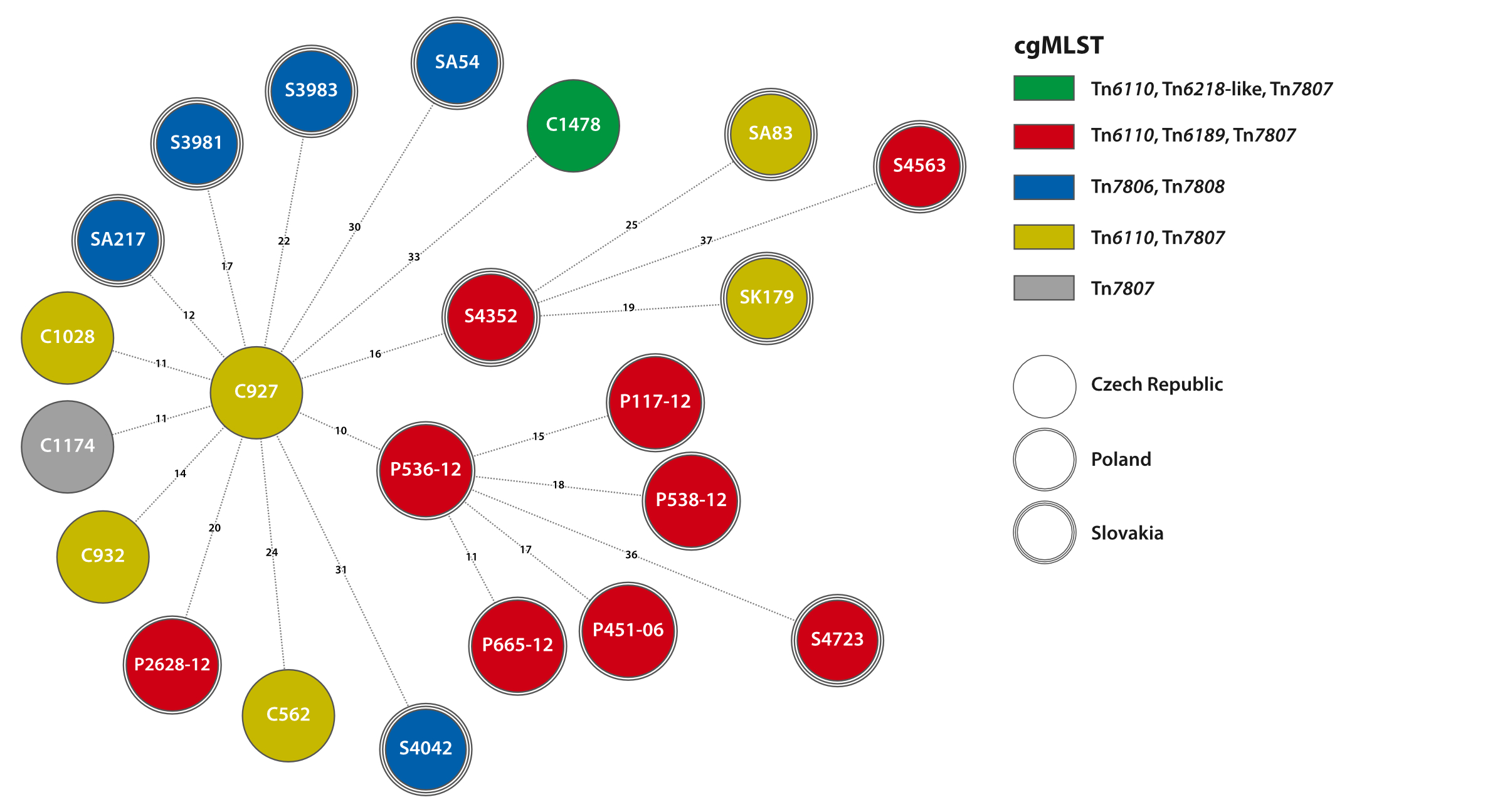

Supplement: FigS2.jpg [file TEMI_A_2482698_SM1064.jpg]

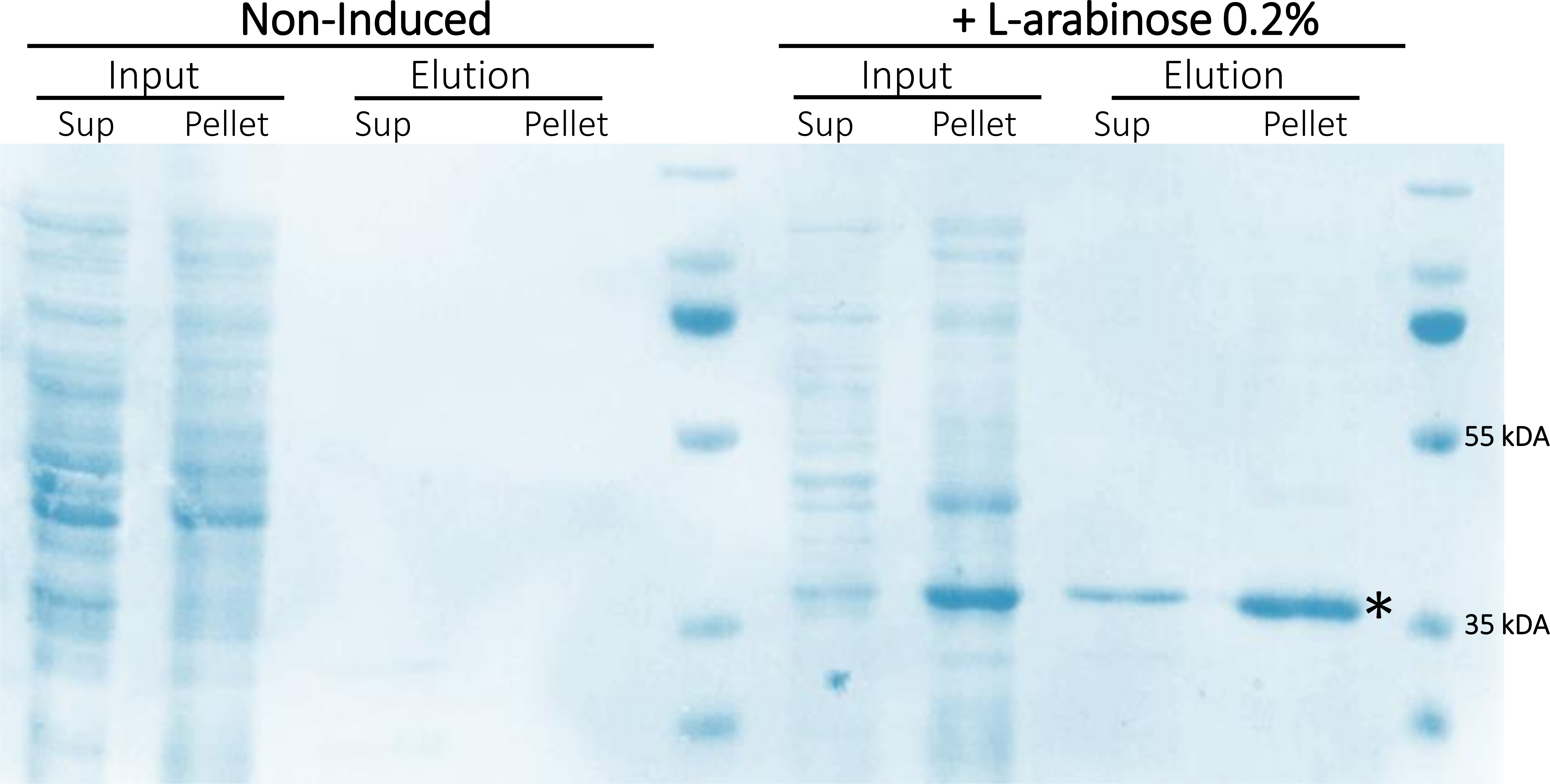

Supplement: FigS3.tiff [file TEMI_A_2482698_SM1063.tiff]
